# Tea Prepared from Dried Cannabis: What Do We Drink?

**DOI:** 10.1021/acs.jafc.4c05940

**Published:** 2024-09-12

**Authors:** Matej Maly, Frantisek Benes, Zuzana Binova, Jana Hajslova

**Affiliations:** Department of Food Analysis and Nutrition, University of Chemistry and Technology, Technická 5, 166 28 Prague 6, Czech Republic

**Keywords:** cannabis tea, bioactive compounds, phytocannabinoids, THC isomers, flavonoids, cannflavins, UHPLC-HRMS/MS

## Abstract

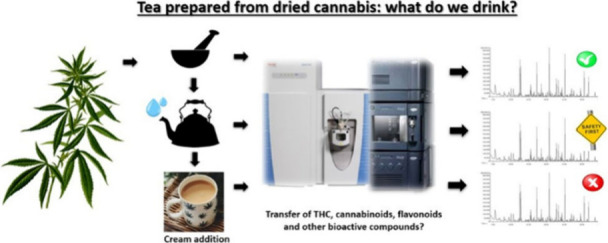

Besides many other uses, dried *Cannabis* may be used for “tea” preparation. This study focused on a comprehensive characterization of an aqueous infusion prepared according to a common practice from three fairly different *Cannabis* cultivars. The transfer of 42 phytocannabinoids and 12 major bioactive compounds (flavonoids) into the infusion was investigated using UHPLC-HRMS/MS. Phytocannabinoid acids were transferred generally in a higher extent compared to their counterparts; in the case of Δ^9^-THC, it was only in the range of 0.4–1.9% of content in the *Cannabis* used. A dramatic increase of phytocannabinoids, mainly of the neutral species, occurred when cream was added during steeping, and the transfer of Δ^9^-THC into “tea” achieved a range of 53–64%. Under such conditions, drinking a 250 mL cup of such tea by a 70 kg person might lead to multiple exceedance of the Acute Reference Dose (ARfD), 1 μg/kg b.w., even in the case when using hemp with a Δ^9^-THC content below 1% in dry weight for preparation.

## Introduction

*Cannabis sativa* L., a medicinal herb first recorded over 5,000 years ago, is rich in pharmacologically active compounds.^1^ In particular, the phytocannabinoid group, with almost 200 variants identified, is particularly interesting because of its interactions with the endocannabinoid system.^2^ This system plays a crucial role in the regulation of cognitive and emotional processes within the human central nervous system, influencing behavior, mood disorders, and neurological conditions like epilepsy.^3,4^ Beyond these unique compounds, numerous other phytochemicals are produced through secondary metabolism in this plant; currently, more than 500 of them have been described, including flavonoids, terpenoids, stilbenoids, and alkaloids.^5,6^ Despite extensive studies, the precise impact of these compounds on the general medicinal effects of *Cannabis* remains under debate.^7−10^ However, some of these compounds are known for their extensive beneficial properties (antioxidant, anti-inflammatory, neuroprotective, etc.), such as typical flavonoids of *Cannabis*, cannflavin A and B, or other flavonoids present in *Cannabis* plants, e.g., vitexin or orientin.^11−13^

*Cannabis* can be consumed in various ways, with the smoking of dry flowers and leaves being the most common. However, healthier alternatives, such as vaporization or oral use, are now recommended by the authorities to cannabis patients.^14^ Most such applications include a heating step (>135 °C) that completely changes the bioactive profile of *Cannabis* by decarboxylation of phytocannabinoid acids.^15^

Among medical patients and recreational users, an increasingly popular way of consuming *Cannabis* is in the form of an aqueous infusion, the so-called cannabis “tea”. In Europe, herbal mixes of *Cannabis* leaves and flowers are widely available for tea preparation and are often considered “healthy”. The boiling point of water (100 °C) and its high polarity lead in addition to (partial) extraction of phytocannabinoids also to coextraction of various other bioactive compounds from the *Cannabis* plant, including antioxidants like flavonoids. Conversely, smoking dry *Cannabis* is often preferred by people seeking the psychoactive effects of THC, which increases due to THCA decarboxylation at burning temperatures. However, this method also exposes users to dangerous byproducts, such as polycyclic aromatic hydrocarbons (PAHs), which are generated through the pyrolysis of plant material.

Despite the growing popularity and availability of cannabis tea, relatively few studies have focused on the transfer of psychotropic and bioactive compounds into such infusions. Early research confirmed the partial transfer of the psychotropic compounds Δ^9^-tetrahydrocannabinol (Δ^9^-THC) and its precursor Δ^9^-tetrahydrocannabinolic acid (Δ^9^-THCA) from *Cannabis* leaves to cannabis tea.^16,17^ The first complex evaluation of the cannabinoid composition of cannabis tea was performed in 2007.^18^ According to these researchers, the influence of various parameters of tea preparation (e.g., volume of water, amount of *Cannabis*, boiling time) on phytocannabinoid transfer does not result in dramatic changes to the composition of tea, either quantitatively or qualitatively. Rather, their findings indicate that the solubility of THC in water at 100 °C is low; therefore, cannabis tea has only limited potency. Their investigation of the transfer of nonpsychotropic phytocannabinoids was limited to qualitative analysis of five of them detected using the HPLC-UV technique.^18^ In another study, the authors analyzed phytocannabinoids in hemp leaves before and after tea preparation.^19^ Although this approach can significantly simplify the analysis, the results provide only an approximation of the true values of valuable and unfavorable compounds in cannabis tea. Furthermore, this study was limited to the six most abundant phytocannabinoids in the variety used in the study.

In the most recent investigations, the cannabinoid profiles and concentrations across 23 different hemp tea samples were analyzed, alongside the examination of the transfer dynamics of 16 cannabinoids from hemp tea into their respective infusions.^20^ This study confirmed the pronounced variability in cannabinoid transfer, which is dependent on the specific composition of *Cannabis.* Furthermore, a relatively small transfer of Δ^9^-THC into cannabis tea was observed (ranking between 0.3 and 2%). It is noteworthy that scientific interest has focused predominantly on the best known phytocannabinoid in *Cannabis* plants, trans-Δ^9^-THC, overlooking its other isomers such as cis-Δ^9^-THC and exo-THC. The scientific literature provides limited information on cis-Δ^9^-THC and exo-THC; however, current knowledge indicates that cis-Δ^9^-THC has a lower affinity for CB1 receptors than its enantiomer, trans-Δ^9^-THC, yet it remains an effective cannabimimetic agent.^21^ Similarly, exo-THC (i.e., Δ^9,11^-THC) is a psychotropic cannabinoid with a potency comparable to that of Δ^8^-THC. Importantly, both cis-Δ^9^-THC and exo-THC are found in significantly higher amounts in CBD-dominant strains of *Cannabis* (chemotypes III^22^), which are frequently chosen for hemp tea preparations.^23,24^ This study is the first to consider not only the transfer of trans-Δ^9^-THC but also its less known isomers—cis-Δ^9^-THC and exo-THC—from dried cannabis to cannabis tea.

In the context of cannabis tea preparations, there has been a notable inclination to introduce full cream milk or cream during the aqueous infusion of dried C*annabis*, which is occasionally advocated by producers and vendors of cannabis tea. Despite its prevalence, scientific investigation of this practice remains very limited. A single study in this domain indicated that cream inclusion was inversely correlated with cannabinoid transfer.^25^ Contrary to these observations, our analytical findings show a notable augmentation in the transfer of mostly nonpolar phytocannabinoids because of the introduction of lipidic matrices into aqueous infusion systems.

Overall, the literature shows that important results have been obtained as far as the transfer of THC from the *Cannabis* plant to cannabis tea is concerned. However, no comprehensive study on the transfer of other bioactive compounds to cannabis tea has yet been conducted. Furthermore, the addition of fatty matrices, such as milk or cream, to the aqueous infusion during the steeping of cannabis tea was not considered, although some producers recommend this practice.

Here, we characterize three different varieties of *Cannabis* (chemotypes I and III^22^), their aqueous infusions, and their aqueous infusions with cream addition by UHPLC-HRMS target analysis involving 42 phytocannabinoids and 12 flavonoids that occur in plants of *Cannabis sativa* L. In addition, we present their more complex characterization by target screening against our in-house spectral library, which includes more than 700 secondary metabolites identified in *Cannabis* plants.

## Materials and Methods

### Chemicals and Materials

LC-MS grade chemicals (acetonitrile, ammonium formate, formic acid, and acetic acid) were purchased from Merck (Germany). Deionized water (18 mΩ) was obtained from a Milli-Q system (Merck Millipore, USA). P.A. 96% ethanol and P.A. methanol were purchased from Lach-Ner (Czech Republic). Analytical standards of 42 phytocannabinoids: canabidiolic acid (CBDA), cannabidiol (CBD), delta-9-tetrahydrocannabidiolic acid A (Δ^9^-THCA-A), trans-delta-9-tetrahydrocannabinol (trans-Δ^9^-THC), cis-delta-9-tetrahydrocannabinol (cis-Δ^9^-THC), delta-8-tetrahydrocannabinol (Δ^8^-THC), cannabinolic acid (CBNA), cannabinol (CBN), cannabinodiol (CBND), cannabigerolic acid (CBGA), cannabigerol (CBG), cannabidivarinic acid (CBDVA), cannabidivarin (CBDV), delta-9-tetrahydrocannabidivarinic acid (Δ^9^-THCVA), delta-9-tetrahydrocannabidivarin (Δ^9^-THCV), delta-8-tetrahydrocannabidivarin (Δ^8^-THCV), cannabichromevarinic acid (CBCVA), cannabichromevarin (CBCV), cannabichromenic acid (CBCA), cannabichromen (CBC), cannabicyclolic acid (CBLA), cannabicyclol (CBL), cannabielsoin (CBE), cannabicitran (CBT), cannabigerovarinic acid (CBGVA), cannabigerovarin (CBGV), cannabivarinic acid (CBVA), cannabivarin (CBV), cannabidibutol (CBDB), delta-9-tetrahydrocannabibutol (Δ^9^-THCB), cannabigerobutol (CBGB), cannabidihexol (CBDH), delta-9-tetrahydrocannabiphorol (Δ^9^-THCP), cannabichromeorcin (CBCO), cannabinol monomethyl ether (CBNM), cannabigerol monomethyl ether (CBGM), cannabigerorcin (CBGO), cannabigerorcinic acid (CBGOA), cannabigerol quinone acid (CBGAQ), cannabinol methyl ether (CBNME), and 10 internal isotopically labeled standards (Δ^9^-THC-D3, Δ^8^-THC-D3, CBD-D3, CBN-D3, Δ^9^-THCA-D3, CBG-D3, CBGA-D3, CBDA-D3, CBCA-D3, and CBC-D3) were supplied by Cerilliant Corporation (USA) and Cayman Chemical (USA), and their purity was in the range of 95.0%–99.8%. The Cannabis Flavonoids mixture, consisting of analytical standards of 12 flavonoids: orientin, vitexin, isovitexin, myricetin, quercetin, isoquercetin, luteolin, kaempferol, apigenin, chrysoeriol, cannflavin A, and cannflavin B, was supplied by Cayman Chemical (USA).

### Sample Information

Samples of dried plant material were selected based on their distinct chemical profiles. The hemp variety ‘Tisza’ (chemotype III), characterized by a low phytocannabinoid content and marketed as hemp tea, along with ’CBD Therapy’ (chemotype III), known for its high levels of CBD, were provided by a cannabis tea producer (Hempoint Company, Czech Republic). Euforia (chemotype I), a drug-type *Cannabis* intended for medical patients with a prescription, was obtained within the research project focused on the impact of growing conditions on the phytocannabinoids profile. The dried plant material consisted of ground inflorescences. Before analysis, all samples were stored at room temperature and protected from sunlight.

### UHPLC-HRMS/MS Method

#### Target Analysis of Phytocannabinoids and Flavonoids

Quantitative analysis of phytocannabinoids was performed by using the ISO 17025 accredited UHPLC-HRMS/MS method. Sample components were separated using an UltiMate 3000 liquid chromatograph (Thermo Scientific, USA) equipped with a reverse phase column, Acquity UPLC BEH C18 (150 mm × 2.1 mm; 1.7 μm, Waters, USA). Mobile phases consisted of (A) water–acetonitrile (95:5, v/v) with 15 mM ammonium formate and 0.2% formic acid and (B) acetonitrile–water (95:5, v/v) with 5 mM ammonium formate and 0.2% formic acid. The total run time of the method was 19 min, the flow rate was 0.3 mL/min, and the injection volume was 3 μL. The multistep gradient program started with 5% B, increased to 70% B until 1 min, and then to 80% B until 13 min. This was followed by a rapid change to 100% B (in 0.5 min), holding at 100% B for 3 min, and returning to the initial conditions for 2.5 min.

Quantitative analysis of flavonoids was performed using an UltiMate 3000 liquid chromatograph (Thermo Scientific, USA) equipped with a reverse phase column, Acquity UPLC BEH C18 (150 mm × 2.1 mm; 1.7 μm, Waters, USA). Mobile phases consisted of (A) water–methanol (95:5, v/v) with 5 mM ammonium formate and 0.1% formic acid and (B) 2-propanol-methanol–water (65:30:5, v/v/v) with 5 mM ammonium formate and 0.1% formic acid. The total run time of the method was 18.5 min, the flow rate was 0.3 mL/min, and the injection volume was 3 μL. The multistep gradient program started with 5% B, increased to 25% B until 1 min, and then to 100% B until 12 min. This was followed by a 4 min hold at 100% B and return to the initial conditions for 2.5 min.

To detect the targeted analytes, a Q-Exactive Plus orbital trap mass spectrometer (Thermo Scientific, USA) was employed. The positive/negative electrospray ionization (ESI ± ) parameters (ESI) were as follows: sheath/aux gas (N_2_) flow 45/10 arb. u., aux gas temperature 300 °C, spray voltage 3.5 kV, and S-lens RF level 55. The detector operated in two acquisition modes: Full scan MS and Parallel Reaction Monitoring (PRM). Detection conditions were as follows: full scan MS: resolution 70 000 full width at half-maximum (fwhm), scan range 200–1 000 *m*/*z*, automatic gain control (AGC) target 2e5, maximum inject time (maxIT) 50 ms, and for PRM: resolution 17 500 fwhm, scan range *m*/*z* 50 - *m*/*z* of fragmented analyte (+ 25 *m*/*z*), AGC target 2e5, maxIT 50 ms, isolation window width 1 *m*/*z*, normalized collision energy (NCE) 28, 35, and 42%. The exact masses of the target analytes (for both phytocannabinoids and flavonoids) and their fragment ions are summarized in Supporting Table S1. For calculation, instrumental analysis, and data processing, Xcalibur 4.0 (Thermo Scientific) was used.

The quantification of phytocannabinoids in the samples was performed using a set of solvent calibration standards (in ethanol) containing all 42 phytocannabinoids, and a mixture of 10 internal standards was added to each calibration point at 30 ng/mL. For all analytes, the limits of quantification (LOQ), which represent the lowest calibration points, were in the range of 0.50–1 mg/kg, and the calibration curves were linear up to 50 mg/kg (coefficient of determination, *R*^2^, ≥0.999). The relative standard deviation (RSD) of the replicate sample analysis was 6–12%.

The quantification of flavonoids in the samples was performed using a set of solvent calibration standards (in 80% aqueous methanol) containing 12 flavonoids. For all analytes, the limits of quantification (LOQ) represented the lowest calibration point, 1 mg/kg, and the calibration curves were linear up to 50 mg/kg (coefficient of determination, *R*^2^, ≥ 0.999). The relative standard deviation (RSD) of the replication sample analysis was 2–8%.

#### Target Screening of Other Phytocannabinoids and Bioactive Compounds

The target screening was performed based on the procedure described in our earlier publication.^26^ Chromatographic separation was performed using an Agilent 1290 Infinity LC system, an ultrahigh performance liquid chromatograph (Agilent Technologies, USA). The system was equipped with an Acquity UPLC BEH C18 analytical column (150 mm × 2.1 mm; 1.7 μm; Waters, USA). The mobile phases consisted of (A) water–acetonitrile (95:5, v/v) with 5 mM ammonium acetate and 0.1% acetic acid, and (B) 2-propanol-acetonitrile–water (75:20:5, v/v/v) with 5 mM ammonium acetate and 0.1% acetic acid. The total run time of the method was 19 min, and the injection volume was 3 μL.

The gradient program started with 0% B, increased linearly to 65% until 4.0 min, and then to 77.5% B in 4 min. This was followed by a change to 100% B in 5 min, holding at 100% B for 5 min, and returning to the initial conditions for 2 min.

The detector used for the targeted screening was a 6560 Ion Mobility Q-TOF MS mass spectrometer (Agilent Technologies, USA). The AJS ion source operated in positive (ESI+) and negative (ESI-) ionization modes with the following settings: jet voltage 400 V, capillary voltage 3.5 kV, nebulizer pressure 40 psi (ESI+)/25 psi (ESI-), drying gas (N_2_) temperature 280 °C (ESI+)/300 °C (ESI-), drying gas flow 12 L/min, sheath gas (N_2_) temperature 350 °C (ESI+)/370 °C (ESI-), and sheath gas flow 12 L/min. The mass spectrometer was operated in Auto MS/MS mode with the following parameter settings: mass range 100–1,000 *m*/*z*, acquisition rate 3 spectra/s (MS) and 12 spectra/s (MS/MS), and collision energy 20 eV.

The targeted screening of compounds for which analytical standards were not available was performed using an “in-house” spectral library created using Agilent MassHunter PCDL (Pharmacologically Active Compounds Database Library) SW. The library contained 281 phytocannabinoids (in addition to the 42 phytocannabinoids analyzed by the quantitative method) and 452 other bioactive compounds (in addition to the 12 flavonoids analyzed by the quantitative method), including flavonoids, phenolics, and others reported to occur in *Cannabis*.^27−36^ The library involved a molecular formula (elemental composition) for the extraction of targeted features using Agilent MassHunter Profinder 8.0 SW. The parameters used for the targeted screening were as follows: targeted ions [M + H]^+^ and [M + NH_4_]^+^ (ESI+)/[M – H]^−^ and [M + CH_3_COO]^−^ (ESI-), exact mass match tolerance <5 ppm, score threshold 90%, and height threshold 5000 counts.

The identity of the detected compounds was confirmed by comparing the experimental and *in silico* MS/MS fragmentation spectra (using the Agilent MassHunter Molecular Structure Correlator SW). The agreement between the experimental and theoretical spectra was expressed as a correlation score, which represents the degree of agreement with the predicted identity. A correlation score threshold of 70% was established.

### Sample Preparation

#### Dry Plant Material

For phytocannabinoids analysis, dry plant material was processed according to the procedure described in our earlier publication.^37^ A homogenized sample (0.5 g) was extracted with 2 × 20 mL of ethanol in a 50 mL PTFE centrifuge tube (50 mL – Kartelllabware, IT) using a homogenizer (SPEX SamplePrep, 2010 Geno/Grinder, USA) for 5 min at 1 000 strokes/min. After centrifugation (5 min, 11 180 RCF, Hettich, DE) and filtration, the volume of the combined extracts was adjusted to 50 mL with ethanol. Before analysis, the final extract had to be significantly diluted with ethanol (in ratios of 1:9, 1:99, 1:999, 1:9 999, v/v) to avoid running of some target analytes out of the detector linear range. A mixture of 10 isotopically labeled internal standards was added to the diluted extract to compensate for matrix effects. Regardless of the dilution, the concentration of each internal standard was 30 ng/mL.

For qualitative target screening of other phytocannabinoids and bioactive compounds, a nondiluted final ethanolic extract was used.

For flavonoid analysis, the following procedure was used: 1 g of homogenized sample was extracted with 3 × 20 mL of aqueous methanol (80%) in a 50 mL PTFE centrifuge tube (50 mL – Kartelllabware, IT) using a homogenizer (SPEX SamplePrep, 2010 Geno/Grinder, USA) for 5 min at 1 000 strokes/min. After centrifugation (5 min, 11 180 RCF, Hettich, DE) and filtration, the volume of the combined extracts was adjusted to 100 mL with aqueous methanol (80%). Before analysis, the final extract had to be significantly diluted with aqueous methanol (in ratios of 1:9, 1:99, and 1:999 v/v) to avoid running some target analytes out of the detector linear range.

#### Cannabis “Teas”

Dry herbal material was used to prepare the respective decoctions. To 1 g of homogenized cannabis sample filled in a nonwoven commercial tea bag was added 250 mL of distilled boiling water, and the boiling was continued for 10 min to maximize the extraction of phytocannabinoids. After 10 min, the tea bag was removed, and the decoction was filtered into a 250 mL volumetric flask. The volume of the cooled decoction was then adjusted with distilled water. This procedure was performed in triplicate, and the reported concentrations of phytocannabinoids and flavonoids are the mean values of these three measurements. To concentrate the sample, 5 mL of decoction was mixed with 27 mL of acetonitrile (azeotropic mixture facilitated evaporation), and the residue was then dissolved in 2 mL of ethanol (for phytocannabinoid analysis) and 2 mL of 80% methanol (for flavonoid analysis). Before analysis, the concentrated sample for phytocannabinoid analysis was diluted with ethanol to avoid running of the analytes at a higher concentration out of the detector range (in ratios of 1:9 and 1:99, v/v) with the addition of a mixture of 10 isotopically labeled internal standards of the same concentration (30 ng/mL) for every dilution. The sample for the analysis of flavonoids was diluted with aqueous methanol (80%) in ratios of 1:9 and 1:99, v/v.

#### Cannabis “Teas” with Added Cream

To the cannabis teas prepared as described above was added 20 g of cream with 10% fat. From the 250 mL volumetric flask, an aliquot of 1 mL was taken and diluted with 9 mL of ethanol (for phytocannabinoid analysis)/aqueous methanol (80%) (for flavonoid analysis) for protein precipitation from cream, and the sample was centrifuged (5 min, 11 180 RCF, Hettich, DE) and filtered. Before analysis, the sample for phytocannabinoid analysis was diluted with ethanol to avoid running the analytes at a higher concentration out of the detector range (in ratios of 1:9, 1:99, and 1:999 v/v) with the addition of a mixture of 10 isotopically labeled internal standards at the same concentration (30 ng/mL) for every dilution. The sample for the analysis of flavonoids was diluted with aqueous methanol (80%) in ratios of 1:9 and 1:99, v/v.

For qualitative target screening of other phytocannabinoids and bioactive compounds, a 10-fold diluted sample with ethanol was used. To ensure uniform matrix effects and thus enable a direct comparison between cannabis tea with and without cream addition, an identical amount of cream (20 g) was introduced to aqueous cannabis tea after decoction and removal of cannabis material. Subsequently, both sets of samples underwent the same processing steps, including a 10-fold dilution with ethanol filtered to remove precipitated proteins.

## Results and Discussion

### Phytocannabinoid and Flavonoid Contents in Dried Plant Material

The results of the analysis of phytocannabinoids in the dry *Cannabis* samples are presented in Table 1. Of the 42 targeted phytocannabinoids, 37 were identified in at least one sample. The phytocannabinoid profiles were significantly different, reflecting the distinct chemotypes and varieties of *Cannabis* used in this study (Euforia, chemotype I - Δ^9^-THC/CBD ≫1; Tisza and CBD Therapy, chemotype III - Δ^9^-THC/CBD ≪1^22^). The Δ^9^-THC and Δ^9^-THCA concentrations in the Tisza and CBD Therapy samples complied with the legal limits of the European Union for technical *Cannabis* (<0.3% in dry plant material^38^). On the other hand, in the Euforia variety, which represents medical-grade *Cannabis,* the content of Δ^9^-THC and Δ^9^-THCA was as high as 14.2% (w/w). Such *Cannabis* is available for prescription in territories where its medical application is authorized. Given the assumption that tea represents one of the methods for the administration of medical-grade *Cannabis* to patients, our research also focused on chemotype I plant.

**Table 1:** Concentration of Phytocannabinoids (mg/kg) in the Three Tested Cannabis Varieties

	mg/kg		
Phytocannabinoid^a^	Euforia	CBD therapy	Tisza
CBDA	4 169 ± 417	8 815 ± 882	5 079 ± 508
Δ^9^-THCA	113 432 ± 11 343	17 ± 4.3	130 ± 20
CBD	5 067 ± 507	58 764 ± 5 876	11 005 ± 1 101
trans-Δ^9^-THC	28 801 ± 2 880	1 053 ± 105	547 ± 82
cis-Δ^9^-THC	135 ± 20	666 ± 100	145 ± 22
exo-THC	196 ± 29	317 ± 48	29 ± 5.8
CBGOA	0.55 ± 0.22	<0.5	0.95 ± 0.38
CBDVA	22 ± 4.4	61 ± 9.2	64 ± 9.6
CBGVA	3.8 ± 1.3	<0.5	<0.5
CBGV	1.5 ± 0.5	<0.5	<0.5
CBDV	21 ± 4.2	270 ± 41	97 ± 15
CBGB	2.1 ± 0.7	<0.5	<0.5
CBDB	10 ± 2.5	117 ± 18	23 ± 4.6
CBE	133 ± 20	310 ± 47	141 ± 21
CBND	<0.5	17 ± 4.3	21 ± 5.3
CBCO	10 ± 2.5	8.2 ± 2.9	<0.5
CBV	95 ± 14	3.3 ± 1.2	1.7 ± 0.6
CBGA	3 535 ± 354	187 ± 28	190 ± 29
CBVA	39 ± 7.8	<0.5	<0.5
CBG	3 155 ± 316	937 ± 141	368 ± 55
CBDH	<0.5	12 ± 3.0	2 ± 0.7
Δ^9^-THCV	286 ± 43	6.9 ± 2.4	11 ± 2.8
Δ^9^-THCVA	568 ± 85	<0.5	3.8 ± 1.3
CBCV	16 ± 4.0	17 ± 4.3	9.6 ± 3.4
Δ^9^-THCB	52 ± 7.8	2.2 ± 417	1.7 ± 0.6
CBN	13 603 ± 1 360	570 ± 86	170 ± 26
CBCVA	5 ± 1.8	3.8 ± 1.3	6.8 ± 2.4
CBNA	3 720 ± 372	4.8 ± 1.7	22 ± 4.4
CBDP	1.1 ± 0.4	7.6 ± 2.7	2.3 ± 0.8
CBC	945 ± 142	1827 ± 183	590 ± 89
Δ^9^-THCH	2.5 ± 0.9	17 ± 4.3	<0.5
CBCA	1 346 ± 135	224 ± 34	1 206 ± 121
Δ^9^-THCP	3.1 ± 1.1	<0.5	<0.5
CBGM	5.5 ± 1.9	<0.5	20 ± 4.0
CBTC	447 ± 67	651 ± 98	60 ± 9.0
CBL	<0.5	4.8 ± 1.7	8.4 ± 2.9
CBLA	<0.5	<0.5	49 ± 10
sum of the tested phytocannabinoids	179 828 ± 17 983	74 891 ± 7 489	20 004 ± 2 000

a The concentrations of CBNME, CBGO, Δ^8^-THC, Δ^8^-THCA, and Δ^8^-THCV were below LOQ (0.5 mg/kg) in all three tested Cannabis varieties

It should be noted that earlier studies concerning the transfer of phytocannabinoids during steeping *Cannabis* into tea primarily focused on trans-Δ^9^-THC, and its precursor, Δ^9^-THCA.^18−20^ Our research extends this scope by including two additional THC isomers, cis-Δ^9^-THC and exo-THC, which interact with endocannabinoid receptors similarly to trans-Δ^9^-THC.^23,39^ Quantifying these compounds is therefore important for a comprehensive assessment of the overall psychotropic potential of cannabis products. Consistent with available studies,^2,39,40^ our results confirmed the significant presence of these isomers in the THC profile of chemotype III plants. In the CBD Therapy sample, cis-Δ^9^-THC and exo-THC accounted for 32% and 16%, respectively, of the total content of THC (which includes all THC isomers). The Tisza variety showed lower but still significant proportions of these isomers, with cis-Δ^9^-THC and exo-THC constituting 20% and 4% of the total THC content. In contrast, for the chemotype I plant (Euforia variety), the content of other isomers compared to trans-Δ^9^-THC was negligible (1%).

Regarding flavonoids, as shown in Table 2, 8 of the 12 targeted analytes were quantified in all samples. Prenylflavonoids, cannflavin A and cannflavin B, secondary metabolites with pronounced anti-inflammatory properties,^41,42^ were generally predominant, with their highest concentration observed in the Euforia cultivar. The Tisza cultivar, which contained the highest overall flavonoid content, was characterized by significantly higher concentrations of orientin and vitexin.

**Table 2:** Concentration of Flavonoids (mg/kg) in the Three Tested Cannabis Varieties

	mg/kg		
flavonoid^a^	Euforia	CBD therapy	Tisza
orientin	42 ± 8.4	72 ± 11	400 ± 60
vitexin	22 ± 4.4	28 ± 5.6	107 ± 16
isoquercetin	23 ± 4.6	27 ± 5.4	12 ± 3.0
luteolin	3.8 ± 1.3	3.5 ± 1.2	12 ± 3.0
apigenin	14 ± 3.5	13 ± 3.3	24 ± 6.0
chrysoeriol	9.2 ± 3.2	4 ± 1.4	6.1 ± 2.1
cannflavin B	77 ± 12	94 ± 14	80 ± 12
cannflavin A	258 ± 39	139 ± 21	120 ± 18
sum of the tested flavonoids	450 ± 68	381 ± 57	761 ± 114

a The concentrations of isovitexin, quercetin, myricetin, and kaempferol were below the LOQ (1 mg/kg) in all three tested *Cannabis* varieties.

### Transfer of Phytocannabinoids, Flavonoids, and Other Bioactive Compounds to Cannabis Tea

The transfer of phytocannabinoids and the most important group of bioactive secondary metabolites occurring in *Cannabis*, flavonoids, to cannabis tea was assessed on the basis of their concentrations determined in respective matrices, i.e., the starting raw material (dried *Cannabis*) and final product (cannabis tea), using fully validated methods described in the Materials and Methods. The percentages of bioactive compounds transferred from dried *Cannabis* to cannabis tea are described in detail in Supplementary Tables 2 and 3.

**Figure 1:**
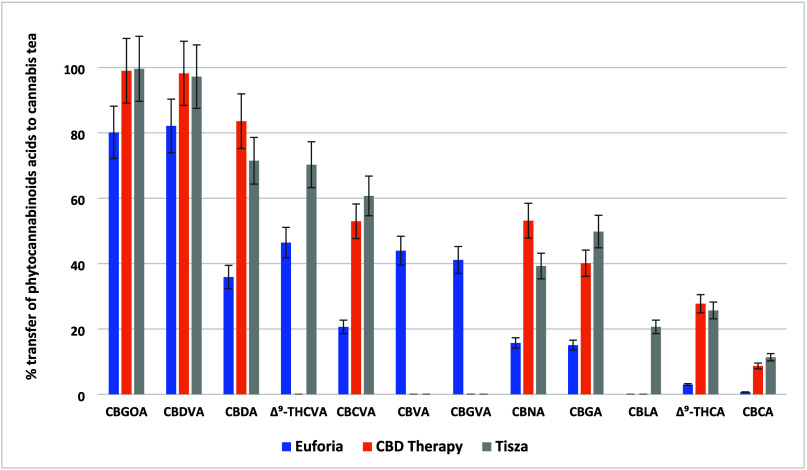
Transfer rates (%) of 12 phytocannabinoid acids from dry *Cannabis* to cannabis tea across the three varieties of *Cannabis*.

**Figure 2:**
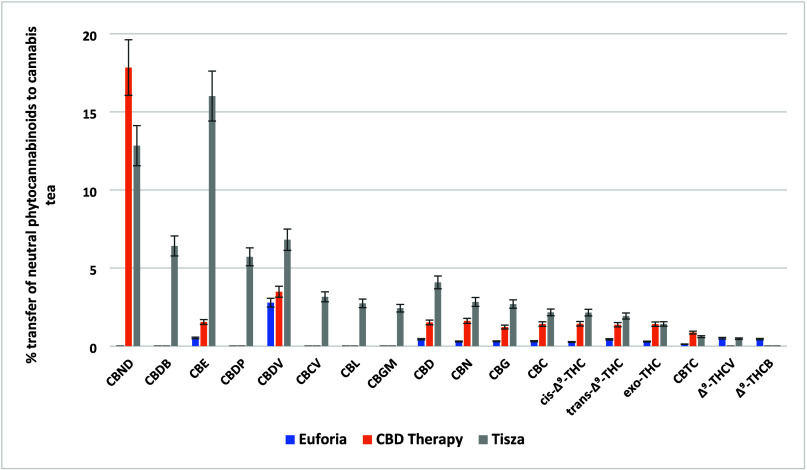
Transfer rates (%) of 18 neutral phytocannabinoids from dry *Cannabis* to cannabis tea in the three varieties of *Cannabis*.

Concerning phytocannabinoids, relatively polar acids such as CBGOA or CBDVA showed the highest transfer rates (80–100%); see Figure 1. Notably, the transfer of CBDA, an important analyte that has attracted considerable scientific interest in recent years due to its potential health benefits,^43,44^ ranged from 71 to 84% in chemotype III varieties, specifically Tisza and CBD Therapy. On the other hand, less polar and neutral phytocannabinoids were transferred to a significantly lower extent; in the case of Δ^9^-THCV or CBCT, it was only around 0.5%; see Figure 2. With regard to THC isomers, the average transfer ranged from only 1% to 1.3%. The data show that information on the composition of *Cannabis* cannot be directly correlated with the phytocannabinoids profile in the prepared cannabis tea. In any case, the transfer of phytocannabinoid acids is significantly higher than that of their neutral counterparts, making cannabis tea an interesting and atypical source of a specific group of phytocannabinoids.

**Figure 3:**
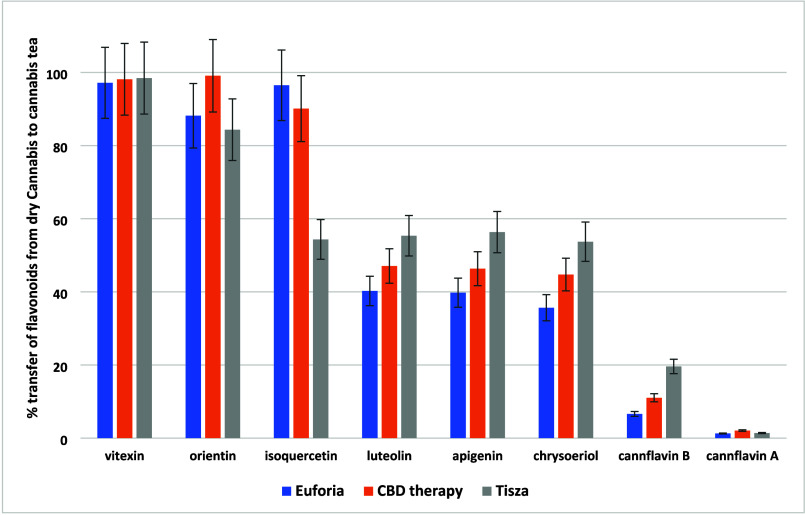
Transfer rates (%) of 8 flavonoids from dry *Cannabis* to cannabis tea in the three varieties of *Cannabis*.

Previous research focusing on phytocannabinoid transfer to aqueous infusions^18−20,25^ involved a narrower scope, analyzing up to 17 analytes compared with the 42 in our study, and did not specifically focus on varieties rich in CBD/CBDA and Δ^9^-THC/Δ^9^-THCA. Furthermore, because *Cannabis* contains other bioactive compounds with positive therapeutic potential besides phytocannabinoids,^33,42,45^ our study is the first to conduct a quantitative analysis of flavonoid transfer into cannabis tea, including prenylflavonoids such as cannflavin A and B. The transfer of flavonoids from dried *Cannabis* to cannabis tea, as illustrated in Figure 3, varied significantly with the polarity of the compounds. For highly polar compounds such as vitexin, orientin, and isoquercetin, the transfer rates were high, ranging on average from 80% to 98%. Semipolar flavonoids such as luteolin, apigenin, and chrysoeriol had transfer rates of approximately 50%, whereas the less polar cannflavins A and B showed lower transfer rates of 1.6% and 12%, respectively.

In addition to the 54 secondary metabolites (42 phytocannabinoids and 12 flavonoids), for which analytical standards were available and thus could be quantified, minor phytocannabinoids and other bioactive compounds (such as terpenoids, flavonoids, stilbenoids, alkaloids, and phenolic amides) that could be present in *Cannabis* plants were searched. An in-house created spectral library involving 733 secondary metabolites identified in *Cannabis sativa* L. plants and reported in the scientific literature^27−36^ was used for target screening of respective accurate *m*/*z* values, both in the positive (protonated molecules) and negative ionization modes (deprotonated molecules). When considering only signals with areas >10e^5^, 220 cannabinoids and 172 non-cannabinoids secondary metabolites (listed in Supplementary Tables 4 and 5) were detected in dried *Cannabis* plant material. The qualitative transfer of these compounds to aqueous infusions is summarized in Table 6. The proportion of compounds detected in both the dry plant material and the aqueous infusion varied between different *Cannabis* samples, ranging from 8% to 14% for cannabinoids and from 36% to 43% for noncannabinoids. These findings underscore that cannabis tea is not only a source of phytocannabinoid acids but also a reservoir of other polar and semipolar non-cannabinoid secondary metabolites with potential beneficial biological activities.

### Transfer of Phytocannabinoids, Flavonoids, and Other Bioactive Compounds to Cannabis Tea with Cream Addition

**Figure 4:**
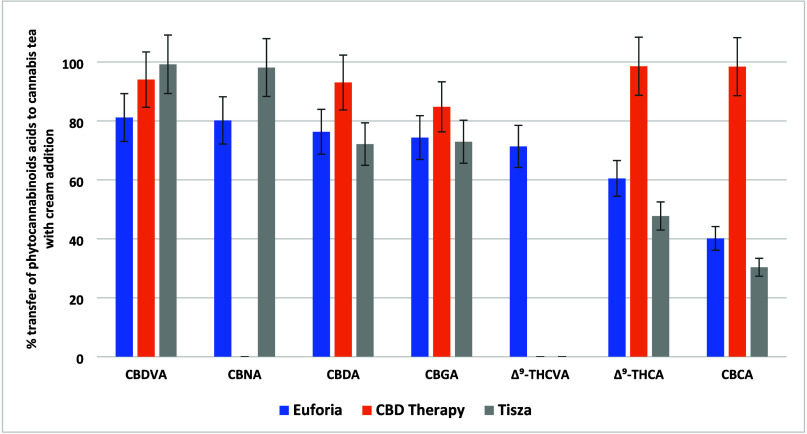
Transfer rates (%) of 7 phytocannabinoid acids from dry *Cannabis* to cannabis tea with cream addition in three varieties of *Cannabis*.

**Figure 5:**
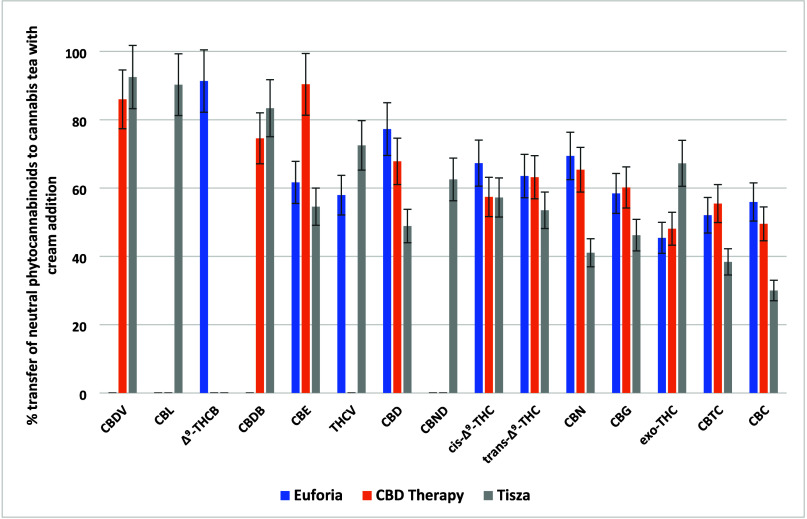
Transfer rates (%) of 15 neutral phytocannabinoids from dry *Cannabis* to cannabis tea with cream addition in three varieties of *Cannabis*.

An important drawback of cannabis tea preparation is the limited solubility of phytocannabinoids in water. Therefore, the impact of solubilizer, such as cyclodextrins, was evaluated.^18^ Although their addition improved the stability and solubility of phytocannabinoids, their use for oral applications remains limited. A more common practice, recommended by some vendors and producers, involves increasing the solubility of phytocannabinoids by adding cream during the preparation of cannabis tea. Interestingly, to date, this method has been investigated in only one study.^25^ Rather surprisingly, the addition of 10% fat to water during the steeping of *Cannabis* was reported to result in a reduced total content of phytocannabinoids in the final drink. The authors explained these results by suggesting the possible chelation between cannabinoid molecules and metal ions (Mg^2+^, Ca^2+^) or alternatively by formation of ester between cannabinoids and the fatty acids present in milk. In contrast to that study, our research demonstrated a dramatic increase in the concentration of phytocannabinoids in cannabis tea with the addition of cream. It should be noted that in our experiments, the addition of 10% fat was 1.6 times higher than that used in prior research,^25^ following producers’ recommendations (see Materials and Methods). As illustrated in Figures 2 and 5 (and Supplementary Tables 2 and 6), this addition primarily enhances the transfer of less polar, neutral phytocannabinoids, while also increasing the transfer rates of phytocannabinoid acids, as shown in Figures 1 and 4. Specifically, the mean transfer rates for neutral phytocannabinoids increased from 4% to 68%, and for phytocannabinoid acids from 46% to 76%. This not only results in a higher total concentration of phytocannabinoids in the cup of tea but also significantly alters the ratio between the neutral and acid forms.

**Figure 6:**
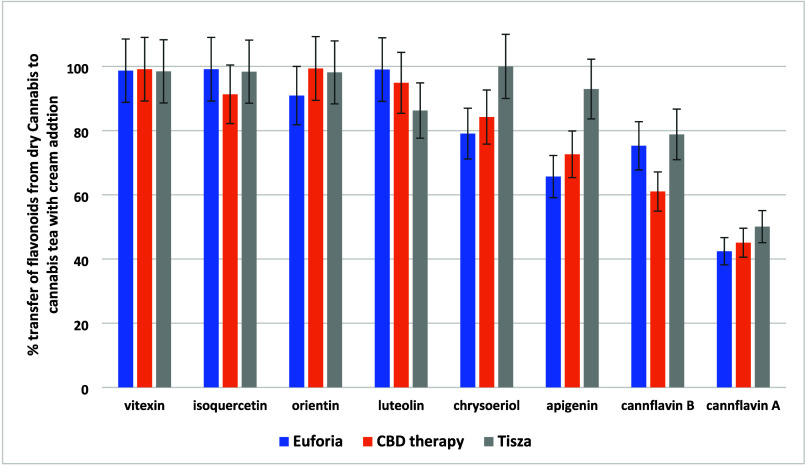
Transfer rates (%) of 8 flavonoids from dry *Cannabis* to cannabis tea with cream addition in three varieties of *Cannabis*.

A similar trend was observed for the transfer of flavonoids, where the average transfer percentage from dry *Cannabis* to cannabis tea increased from 53% to 83% following the addition of cream (compare Figures 3 and 6). This increase was more pronounced for the less polar prenylflavonoids cannflavins A and B, with average transfer percentages rising from 2% to 46% and from 12% to 72%, respectively. Consistent with these findings, Table 6 reveals that adding cream to cannabis tea during decoction resulted in the detection of a broader array of cannabinoid and non-cannabinoid secondary metabolites (with signal areas exceeding 10e5), whereas this trend was more significant for cannabinoid secondary metabolites.

### Consumers’ Exposure through Drinking Cannabis Tea

Table 3 summarizes the exposure of consumers to phytocannabinoids in milligrams per 250 mL of cup for three different *Cannabis* varieties, both with and without cream addition. The increase in total phytocannabinoid content after cream addition is more pronounced in varieties with higher absolute phytocannabinoid concentrations and depends on the initial ratio of acidic to neutral phytocannabinoids in the cannabis product. As illustrated in Table 1, the varieties CBD Therapy and Tisza mainly contained neutral phytocannabinoids, indicating an advanced decarboxylation process. In contrast, the Euforia variety was dominated by acidic cannabinoids, primarily Δ^9^-THCA, likely due to different processing methods, such as drying, storage, and exposure to oxygen and UV radiation.

**Table 3:** Content of Phytocannabinoids in μg in One Cup of Cannabis Tea (250 mL) Prepared from the Tested Varieties, Illustration of the Effect of Added Cream (20 g) on the Extent of Transfer^a^

	Euforia			CBD therapy			Tisza		
	cannabis tea	cannabis tea with cream	*n*-fold increase in concentration	cannabis tea	cannabis tea with cream	*n*-fold increase in concentration	cannabis tea	cannabis tea with cream	*n*-fold increase in concentration
CBDA	1 496 ± 150	3 181 ± 318	2.1	7 366 ± 737	8 201 ± 820	1.1	3 630 ± 363	3 931 ± 393	1.1
Δ^9^-THCA	3 377 ± 338	68 628 ± 6 863	20	3.2 ± 1.1	25 ± 5.0	7.8	33 ± 6.6	79 ± 12	2.4
CBD	22 ± 4.4	3 915 ± 392	178	893 ± 134	39 854 ± 3 985	45	449 ± 67	5 818 ± 582	13
trans-Δ^9^-THC	126 ± 19	18 296 ± 1 830	145	14 ± 3.5	665 ± 100	48	11 ± 2.8	288 ± 29	26
cis-Δ^9^-THC	0.41 ± 0.16	91 ± 14	228	10 ± 2.5	382 ± 57	38	3.1 ± 1.1	52 ± 7.8	17
exo-THC	0.62 ± 0.25	89 ± 13	148	4.5 ± 1.6	152 ± 23	34	0.41 ± 0.16	14 ± 3.5	35
CBGA	532 ± 80	2629 ± 263	4.9	75 ± 11	158 ± 24	2.1	95 ± 14	149 ± 22	1.6
CBNA	585 ± 88	2982 ± 298	5.1	2.5 ± 0.9	NF	-	8.7 ± 3.0	13 ± 3.3	1.5
CBDVA	18 ± 4.5	17 ± 4.3	0.9	58 ± 8.7	57 ± 8.6	1	61 ± 9.2	58 ± 8.7	1
Δ^9^-THCVA	264 ± 40	405 ± 61	1.5	NF	NF	-	1.3 ± 0.46	NF	-
CBGOA	0.41 ± 0.16	NF	-	0.41 ± 0.16	NF	-	1.0 ± 0.35	NF	-
CBGVA	1.5 ± 0.53	NF	-	0.1 ± 0.05	NF	-	1.1 ± 0.39	NF	-
CBVA	17 ± 4.3	NF	-	NF	NF	-	NF	NF	-
CBCA	9.4 ± 3.3	540 ± 81	57	19 ± 4.8	221 ± 33	12	137 ± 21	365 ± 55	2.7
CBLA	NF	NF	-	NF	NF	-	10 ± 2.5	25 ± 5.0	2.5
CBCVA	1.1 ± 0.39	NF	-	2 ± 0.80	NF	-	2.9 ± 1.0	NF	-
CBN	40 ± 8.0	9 441 ± 944	236	9.2 ± 3.2	373 ± 56	41	0.40 ± 0.16	77 ± 12	193
CBC	3.0 ± 1.1	528 ± 79	176	26 ± 5.2	905 ± 136	35	0.90 ± 0.36	231 ± 23	257
CBG	10 ± 2.5	1 843 ± 184	184	11 ± 2.8	564 ± 85	51	10 ± 1.0	195 ± 29	20
CBTC	0.60 ± 0.24	233 ± 35	388	5.7 ± 2.0	361 ± 54	63	NF	15 ± 3.8	-
CBDV	0.62 ± 0.25	NF	-	9.4 ± 3.3	232 ± 35	25	14 ± 3.5	55 ± 8.3	3.9
CBND	NF	NF	-	3.1 ± 1.1	NF	-	2.7 ± 0.90	NF	-
Δ^9^-THCV	1.4 ± 0.49	165 ± 25	118	NF	NF	-	NF	NF	-
CBND	NF	NF	-	3.1 ± 1.1	NF	-	1.2 ± 0.42	13 ± 3.3	11
CBDB	NF	NF	-	4.5 ± 1.6	88 ± 13	20	1.5 ± 0.53	12 ± 3.0	8
CBE	0.68 ± 0.27	82 ± 12	117	4.8 ± 1.7	280 ± 42	58	22 ± 4.4	77 ± 12	3.5
Δ^9^-THCB	0.21 ± 0.08	48 ± 10	240	NF	NF	-	NF	NF	-
Sum	6 506 ± 651	113 114 ± 11 311	17	8 526 ± 853	52 519 ± 5 252	6.2	4 498 ± 450	11 467 ± 1 147	2.5

a NF = not found.

The primary concern for safe consumption of cannabis tea is the risk of unintended intake of psychotropic phytocannabinoids. The European Food Safety Authority (EFSA) in 2015 established an Acute Reference Dose (ARfD) of 1 μg/kg body weight for Δ^9^-THC, serving as a guideline for assessing the risk of cannabis products.^46^Table 5 evaluates the risk of exceeding ARfD for Δ^9^-THC when drinking 250 mL of cannabis teas, with and without cream addition, in the three varieties of *Cannabis.* The minimal transfer of Δ^9^-THC into aqueous infusions implies that only the high-potency Euforia variety would slightly exceed ARfD for a 70 kg individual. For chemotype III varieties, CBD Therapy and Tisza, a 70 kg individual would need to consume more than four and six cups, respectively, prepared according to the producers’ recommendations, to reach ARfD for Δ^9^-THC. These findings are consistent with the conclusions of other studies,^18,20^ indicating that consuming aqueous cannabis tea from chemotype III varieties is relatively safe. However, other Δ^9^-THC isomers, such as cis-Δ^9^-THC and exo-THC, which are often present in chemotype III in significant concentrations, are often overlooked. Including these isomers, the consumption threshold for exceeding ARfD for a 70 kg individual decreases to 0.6/1.25 L of tea prepared from CBD Therapy/Tisza, respectively (see Table 5). Although these isomers probably exhibit a weaker psychotropic effect compared to trans-Δ^9^-THC,^23,39,47^ they remain psychoactive substances. Therefore, its presence, particularly in chemotype III varieties known for their higher concentrations, should not be overlooked.

**Table 4:** Content of Flavonoids in μg in One Cup of Cannabis Tea (250 mL) Prepared from the Tested Varieties, Illustration of the Added Cream (20 g) on the Extent of Transfer

	Euforia			CBD therapy			Tisza		
	cannabis tea	cannabis tea with cream	*n*-fold increase in concentration	cannabis tea	cannabis tea with cream	*n*-fold increase in concentration	cannabis tea	cannabis tea with cream	*n*-fold increase in concentration
orientin	37 ± 7.4	38 ± 7.6	1.0	71 ± 11	71 ± 11	1.0	338 ± 51	392 ± 59	1.2
vitexin	21 ± 4.2	22 ± 4.4	1.0	27 ± 5.3	27 ± 5.4	1.0	106 ± 16	105 ± 16	1.0
isoquercetin	22 ± 4.4	23 ± 4.6	1.0	25 ± 6.3	25 ± 6.4	1.0	6.3 ± 2.2	11 ± 2.8	1.7
luteolin	1.8 ± 0.63	4.4 ± 1.5	2.4	1.9 ± 0.67	3.9 ± 1.4	2.1	6.9 ± 2.4	11 ± 2.8	1.6
apigenin	5.5 ± 1.9	9.1 ± 3.2	1.7	5.8 ± 1.5	9.1 ± 2.3	1.6	13 ± 3.3	22 ± 4.4	1.7
chrysoeriol	3.5 ± 1.2	7.7 ± 2.7	2.2	1.9 ± 0.67	3.6 ± 1.3	1.9	3.5 ± 1.2	6.5 ± 2.3	1.9
cannflavin B	5.1 ± 1.8	58 ± 8.7	11	10 ± 2.5	57 ± 8.6	5.7	16 ± 4.1	63 ± 9.5	3.9
cannflavin A	3.3 ± 0.83	109 ± 27	33	2.9 ± 1.0	63 ± 13	22	1.7 ± 0.60	60 ± 9.0	35
sum	100 ± 15	271 ± 41	2.7	147 ± 22	261 ± 22	1.8	491 ± 74	671 ± 101	1.4

However, the introduction of a hydrophobic component, such as fatty cream, during the decoction process fundamentally alters the phytocannabinoid profile in the drink, leading to a dramatic increase in the concentration of neutral phytocannabinoids, including Δ^9^-THC. This change causes ARfD to be exceeded multiple times after consuming just 250 mL of the infusion for all the three *Cannabis* varieties examined (261 times for Euforia, 9.5 times for CBD Therapy, and 4.1 times for Tisza), as detailed in Table 5.

**Table 5:** Concentration of Δ^9^-THC Isomers in 250 mL cup of Infusion Related to Acute Reference Dose (1 μg/kg Body Weight)

	Euforia		CBD therapy		Tisza	
	aqueous infusion	aqueous infusion with cream addition	aqueous infusion	aqueous infusion with cream addition	aqueous infusion	aqueous infusion with cream addition
trans-Δ^9^-THC (μg/250 mL)	126 ± 19	18 296 ± 1 830	14 ± 3.5	665 ± 100	11 ± 2.8	288 ± 43
% fulfill of ARfD^a^ for 70 kg person after drinking 250 mL of cannabis tea	180%	26 137%	21%	950%	15%	411%
cis-Δ^9^-THC (μg/250 mL)	0.40 ± 0.16	91 ± 14	10 ± 2.5	382 ± 57	3.1 ± 1.1	52 ± 7.8
exo-THC (μg/250 mL)	0.62 ± 0.25	89 ± 13	4.5 ± 1.6	152 ± 23	0.39 ± 0.16	14 ± 3.5
Sum of THC isomers (μg/250 mL)	126 ± 19	18 476 ± 1 848	28 ± 5.6	1 200 ± 120	14 ± 3.5	354 ± 53
% fulfill of ARfD^a^ for 70 kg person after drinking 250 mL of cannabis tea	181%	26 394%	41%	1 714%	20%	506%

a Acute Reference Dose (ARfD) of 1 μg/kg body weight for Δ^9^-THC defined by EFSA in 2015.^46^

**Table 6:** Number and Percentage Transfer of Cannabinoids and Non-Cannabinoids Detected in Three Different *Cannabis* Varieties and in Their Infusions with and without Cream Addition

	Euforia			CBD therapy			Tisza		
	dried plant	aqueous infusion	aqueous infusion with cream addition	dried plant	aqueous infusion	aqueous infusion with cream addition	dried plant	aqueous infusion	aqueous infusion with cream addition
Number of detected cannabinoid secondary metabolites (signal areas >10e^5^)	169	24	31	116	9	12	95	8	14
% transfer from dried C*annabis* to infusion	-	14	18	-	8	10	-	8	15
Number of detected non-cannabinoid secondary metabolites (signal areas >10e^5^)	109	39	40	118	43	43	119	51	55
% transfer from dried C*annabis* to infusion	-	36	37	-	36	36	-	43	46

These results indicate that consuming aqueous cannabis tea from nonpsychotropic varieties (chemotype III) is a generally safe and interesting alternative for people seeking the beneficial effects of *Cannabis* without its psychotropic effects. The boiling point of water is too low to significantly decarboxylate cannabinoid acids into their neutral forms,^20,48^ contrasting with other conventional *Cannabis* consumption methods (e.g., smoking, vaporizing, or baking), which involve thermally induced decarboxylation.^49−51^ Consequently, cannabis tea shows a notable predominance of phytocannabinoid acids and a range of polar to semipolar compounds, including flavonoids and non-cannabinoid secondary metabolites, while the presence of neutral phytocannabinoids is significantly limited. Interestingly, as shown in Table 5, even drinking up to 140 mL of tea prepared from a variety with a high Δ^9^-THC content (chemotype I) under conditions described in the Materials and Methods, would not exceed ARfD for Δ^9^-THC for individuals with a body weight 70 kg.

In summary, our study provides comprehensive insights into the issues associated with the increasing popularity of cannabis tea consumption. By critically evaluating the transfer of 42 phytocannabinoids from three different *Cannabis* varieties (chemotype I and III) to cannabis teas, with a focus on the psychotropic trans-Δ^9^-THC and its lesser-known isomers using UHPLC-HRMS/MS, we conclude that consuming cannabis tea from nonpsychotropic varieties is generally safe and potentially beneficial. We quantified the transfer of 8 key flavonoids, including prenylflavonoids cannflavin A and B, and confirmed the qualitative transfer of numerous other bioactive compounds. Nevertheless, the addition of cream during extraction significantly increases the transfer of neutral phytocannabinoids, including Δ^9^-THC, thereby dramatically altering the safety profile. Consequently, risk assessments of cannabis tea should consider the realistic transfer of phytocannabinoids to the infusion with particular attention to the preparation methods. Furthermore, as cannabis tea represents a unique mode of intake for *Cannabis* bioactive compounds, further investigation into its overall biological effects is needed.

## Supporting Information Available

Table S1: List of 42 quantified phytocannabinoids. Table S2: % transfer of phytocannabinoids from dry *Cannabis* to cannabis tea. Table S3: % transfer of flavonoids from dry *Cannabis* to cannabis tea. Table S4: List of cannabinoids detected in dry *Cannabis* and its infusions. Table S5: List of non-cannabinoid secondary metabolites detected in dry *Cannabis* and its infusions. Table S6: % transfer of phytocannabinoids from dry *Cannabis* to cannabis tea with cream addition. Table S7: % transfer of flavonoids from dry *Cannabis* to cannabis tea with cream addition. Table S8: Mass spectrum and chromatogram of CBDA in standard, dry herb, and aqueous infusion (XLSX)

## Abbreviations Used

ARfD: acute reference dose
CBC: cannabichromen
CBCA: cannabichromenic acid
CBCO: cannabichromeorcin
CBCV: cannabichromevarin
CBCVA: cannabichromevarinic acid
CBD: cannabidiol
CBDA: cannabidiolic acid
CBDB: cannabidibutol
CBDH: cannabidihexol
CBDP: cannabidiphorol
CBDV: cannabidivarin
CBDVA: cannabidivarinic acid
CBE: cannabielsoin
CBG: cannabigerol
CBGA: cannabigerolic acid
CBGAQ: cannabigerol quinone acid
CBGB: cannabigerobutol
CBGOA: cannabigerorcinic acid
CBGV: cannabigerovarin
CBGVA: cannabigerovarinic acid
CBL: cannabicyclol
CBLA: cannabicyclolic acid
CBN: cannabinol
CBNA: cannabinolic acid
CBND: cannabinodiol
CBNME: cannabinol methyl ether
CBT: cannabicitran
CBV: cannabivarin
CBVA: cannabivarinic acid
cis-Δ^9^-THC: cis-delta-9-tetrahydrocannabinol
EFSA: European Food Safety Authority
ESI-: electrospray negative ionization
ESI+: electrospray positive ionization
fwhm: full width at half-maximum
LOQ: limit of quantification
NCE: normalized collision energy
PRM: parallel reaction monitoring
RSD: relative standard deviation
trans-Δ^9^-THC: trans-delta-9-tetrahydrocannabinol
UHPLC-HRMS: ultrahigh-performance liquid chromatography–high-resolution mass spectrometry
UHPLC-HRMS/MS: ultrahigh-performance liquid chromatography–high-resolution tandem mass spectrometry
Δ^8^-THC: delta-8-tetrahydrocannabinol
Δ^8^-THCV: delta-8-tetrahydrocannabidivarin
Δ^9^-THCA-A: delta-9-tetrahydrocannabinolic acid A
Δ^9^-THCB: delta-9-tetrahydrocannabibutol
Δ^9^-THCH: delta-9-tetrahydrocannabihexol
Δ^9^-THCP: delta-9-tetrahydrocannabiphorol
Δ^9^-THCV: delta-9-tetrahydrocannabidivarin
Δ^9^-THCVA: delta-9-tetrahydrocannabidivarinic acid
